# Post-Traumatic Trigeminal Neuropathy: Neurobiology and Pathophysiology

**DOI:** 10.3390/biology13030167

**Published:** 2024-03-04

**Authors:** Tal Eliav, Rafael Benoliel, Olga A. Korczeniewska

**Affiliations:** 1 Medical School for International Health, Faculty of Health Sciences, Ben Gurion University of the Negev, Beer Sheva 8410501, Israel; talzus@post.bgu.ac.il; 2 Center for Orofacial Pain and Temporomandibular Disorders, Department of Diagnostic Sciences, Rutgers School of Dental Medicine, Rutgers, The State University of New Jersey, Room D-837, 110 Bergen Street, Newark, NJ 07101, USA; rafael.benoliel@rutgers.edu

**Keywords:** neuropathic pain, pathophysiology, trauma

## Abstract

**Simple Summary:**

In this article, we provide a review of the current knowledge on trigeminal neuropathy caused of traumatic origin, such as direct nerve damage from dental procedures or motor vehicle accidents. This article focuses on the cellular processes that occur following traumatic nerve damage to the trigeminal nerve, otherwise known as cranial nerve V, that results in chronic pain.

**Abstract:**

Painful traumatic trigeminal neuropathy (PTTN) is a chronic neuropathic pain that may develop following injury to the trigeminal nerve. Etiologies include cranio-orofacial trauma that may result from dental, surgical, or anesthetic procedures or physical trauma, such as a motor vehicle accident. Following nerve injury, there are various mechanisms, including peripheral and central, as well as phenotypic changes and genetic predispositions that may contribute to the development of neuropathic pain. In this article, we review current literature pertaining to the cellular processes that occur following traumatic damage to the trigeminal nerve, also called cranial nerve V, that results in chronic neuropathic pain. We examine the neurobiology and pathophysiology based mostly on pre-clinical animal models of neuropathic/trigeminal pain.

## 1. Introduction

The trigeminal nerve (TN), or fifth cranial nerve, provides sensory–motor function via its three branches: V1, V2, and V3. It transmits information regarding temperature, pain, and touch in the face and provides motor function mainly to the masticatory muscles. When there is a noxious stimulus in the TN region, sensory afferents composed of myelinated Aδ-fibers and non-myelinated C-fibers transmit that information to the primary somatosensory cortex and limbic system [1].

When the nerve is injured, it may manifest on a spectrum of symptoms from no pain to tingling to attacks of severe pain. Trigeminal neuralgia may arise from etiologies including vascular compression, space-occupying lesions, multiple sclerosis, and trauma. When trauma causes injury to the sensory components of the TN resulting in pain, it is termed post-traumatic trigeminal neuropathy (PTTN), a diagnosis of exclusion, with a prevalence of 1.55 to 3.3% [2,3,4].

While neuropathic pain is generally widely researched, the understanding of mechanisms contributing to the development and maintenance of trigeminal neuropathic pain relies heavily on animal studies that focus on spinal, rather than TN, injury. Here we summarize findings regarding first the pathophysiology of PTTN, and then its neurobiology.

## 2. Pathophysiology

Trauma to the TN can be caused by cranio- or orofacial trauma, such as motor vehicle accidents, dental procedures (endodontic treatment, molar extractions), or other surgeries (Caldwell–Luc approach, rhizotomies, nerve blocks). Other injuries may include implant therapy and local anesthetic injections; however, iatrogenic injuries rarely result in chronic neuropathic pain.

While the exact mechanism of PTTN is not entirely understood, we will attempt to summarize current literature. The majority of evidence comes from animal models of neuropathic pain that study injury to the spinal nerve. The cascade of events from peripheral to central in the nervous system result in major changes to neurons and their environment and may be affected by genetic variations [5,6].

## 3. Peripheral Mechanisms

**Figure 1:**
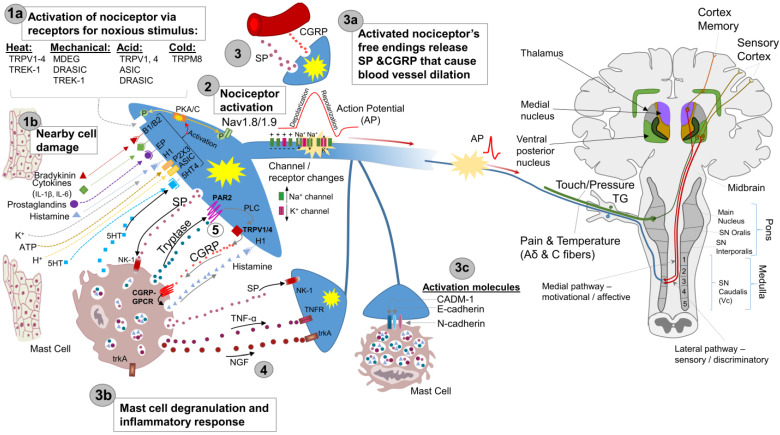
Peripheral sensitization can be initiated via (1a) noxious stimulus or (1b) the release of substances following cell damage. (2) Nociceptors transduce these noxious stimuli (thermal, mechanical, or chemical) into an electric potential resulting in their sensitization. (3) Peripheral sensitization decreases the threshold of electrical activation. (3a) With the release of substances P and CGRP, the inflammatory response is activated, resulting in blood vessel dilation and (3b) mast cell degranulation. (3c) Cell adhesion molecules mediate communication between neurons and mast cells. (4) Mast cells are now activated, releasing nerve growth factor (NGF), tumor necrosis factor alpha (TNF-α), SP, and tryptase, which bind to their respective receptors, which further sensitizes the nerve cell ending. NGF induces growth while TNF-α decreases firing of the neuron. The binding of tryptase induces the release of CGRP, which then binds the G-protein coupled receptor, resulting in the release of histamine. Histamine then binds its receptor, further activating nociceptors and promoting swelling. Figure reprinted with permission from author’s previous publication.

Generally, nerve damage results in altered neuronal electrical activity, peripheral sensitization, and nociceptor activation. The release of inflammatory mediators leads to reduced thresholds and therefore increased excitability of the peripheral terminal membrane, as outlined in Figure 1. The inflammatory response then causes blood vessel dilation, recruitment of white blood cells, and mast cell degranulation, which further decreases the threshold. This leads to swelling and further nociceptor activation that is transmitted through pain and temperature fibers to the cortex. However, in neuropathic pain, this process is dysregulated, manifesting as changes to membrane composition and ion flow, causing an amplified and cyclical response.

In neuropathic pain, patients may feel increased amounts of pain from noxious stimuli or even pain from non-painful stimuli, termed hyperalgesia and allodynia, respectively. This atypical pain is a manifestation of peripheral sensitization or increased responsiveness of nociceptors and decreased thresholds.

## 4. Chemokines and Cytokines

Several pre-clinical studies have investigated the roles of cytokines and chemokines, such as CCL2/receptor CCR2 [7], CXCL13/CXCR5 [8], CXCL2 [9], and CXCL10/CXCR3 [10], in neuropathic pain. Animal studies have shown that overexpression of CCL2/CCR2 is found in the medullary dorsal horn in a model of chronic neuropathic pain. Furthermore, CCR2 antagonists have been found to inhibit nociceptive signaling [11]. Intrathecal injection of CXCL13, known to be upregulated in the spinal cord following spinal nerve ligation [12], has been found to induce mechanical allodynia and increase the activation of ERK and production of TNF-α and IL-1β. Conversely, inhibition of CXCL13 and CXCR5 decreases mechanical allodynia. Similarly, CXCL10 has been found to induce pain hypersensitivity in wild-type mice, but not those lacking CXCR3, and CXCR3 is elevated in mice with chronic constriction injury. CXCL2 is upregulated in pre-clinical studies of injured sciatic nerves [13]. It has been further found to be increased in animals following induction of chronic neuropathic pain via infraorbital nerve constriction [9], and consequently, anti-CXCL2 injection into the trigeminal ganglion decreases pain behavior.

In the realm of pre-clinical models investigating chronic pain, the CX3CL1/R1 signaling pathway plays a pivotal role in modulating nociceptive signaling within the central nervous system (CNS) [14]. The initial modulation occurs at the synapse between primary afferents and dorsal horns in the spinal cord. Notably, following peripheral nerve injury, the pronociceptive sFKN/CX3CR1 pathway demonstrates a significant impact, leading to thermal and mechanical hypersensitivity. This effect is attributed to the induction of intracellular phosphorylation of microglial p38 MAPK, ultimately resulting in the release of pro-inflammatory cytokines IL-1β, IL-6, and NO. Chronic pain models consistently reveal an elevation in both sFKN and CX3CR levels. Moreover, in diverse preclinical models, the absence of CXCR1, as evidenced by CXCR1 knockout mice, consistently correlates with deficits in both traumatic and nontraumatic chronic pain, aligning with reduced microglial activity. These findings collectively underscore the critical involvement of the CX3CL1/R1 signaling pathway in the pathogenesis of chronic pain and emphasize its potential as a therapeutic target for intervention.

## 5. Ion Channels

Current evidence is expanding that ion channels, including sodium, calcium, and potassium channels, play a critical role in the development of pain in the trigeminal system. Many changes result in an increased firing of action potentials. For example, phosphorylation of both tetrodotoxin-resistant voltage-gated sodium channels, Nav1.8 and Nav1.9, decreases the activation threshold and increases excitability. The tetrodotoxin-sensitive sodium channels, Nav1.7 and Nav1.3, have been found to be dysregulated in contrasting directions. In patients with trigeminal neuralgia, Nav1.7, has been found to be downregulated, while Nav1.3, a reducer of nociceptive threshold, is upregulated. Interestingly, Nav1.3 is highly expressed during the embryonic period and decreases to low levels after birth [15]; however, it can upregulate upon nerve injury, increasing excitability, resulting in pain [16].

Calcium-activated potassium channel expression has been found to be decreased following infraorbital nerve ligation, and an agonist of the channel results in increased pain threshold (i.e., mechanical allodynia) [17]. A preclinical model of orofacial pain has found that nerve injury downregulates the voltage-gated potassium channel, Kv7.2 [18], and that downregulation results in increased excitability of the trigeminal ganglia neurons and induced trigeminal neuralgia-like behavior [19].

Dozens of variants for voltage-gated channels have been discovered. Recent studies have found missense mutations in both genes that encode the pore forming α1 subunit of the voltage-gated calcium channels, Cav3.1 and 3.2, resulting in gain of function and contributing to chronic pain [20,21]. Animal models of neuropathic pain involving chronic constriction injury of the infraorbital nerve have demonstrated that non-coding RNAs are involved in the development of trigeminal pain [6,22]. Following nerve injury, micro RNAs are downregulated in the dorsal root ganglion, which is thought to increase pain [23]. These miRNAs have been shown to target Nav1.7 [24]. In a study that upregulated miR-182, Nav1.7 was inhibited, reducing excitability and alleviating pain [25]. Micro RNAS target other voltage-gated sodium channels [26], as well as potassium [27] and calcium [28] channels, with downstream effects on neuropathic pain.

## 6. Ectopic Activity and Spontaneous Pain

Neuropathic pain is characterized by a phenomenon known as ectopic activity, in which spontaneous activity arises along axons within nociceptive pathways and cell bodies in sensory ganglia. This spontaneous activity occurs when a segment of the axon or cell body becomes excessively excitable, leading to the generation of spontaneous action potentials (SAs) [29,30,31].

There are two distinct forms of spontaneous ectopic activity: type 1, which involves subthreshold membrane potential oscillation [31], and type II, which lacks such oscillations. In the ganglia, there are two types of conductance: a voltage-dependent sodium conductance that is physically activating and a voltage-independent conductance attributable to potassium leak. It is proposed that these conductances generate membrane potential oscillations, and when oscillation sinusoids reach threshold amplitude, ectopic firing occurs [32]. Damage to nerves leads to alterations in ion channels, affecting aspects such as their expression, movement, or functionality. This can result in heightened persistent sodium currents or reduced potassium leak currents, both active in the vicinity of the resting potential. These changes may contribute to the relative depolarization of the resting potential, which is often linked with spontaneous activity [33]. Furthermore, hyperpolarizing cationic currents, specifically I_h_, play a role in enhancing the excitability of sensitized peripheral fibers. The I_h_ current density and rate of firing increases in the sensory ganglia and trigeminal ganglion neurons in peripheral nerve injury [34,35,36,37]; blocking of these currents reduces mechanical allodynia and ectopic discharge following nerve injury [35,38].

In cases where there is inflammation surrounding a nerve without any damage to the nerve’s axon, there is an increase in spontaneous activity, triggering heightened mechanosensitivity, which may serve as a source of pain. Prolonged inflammation, if sustained, may potentially result in subsequent damage to the nerve. This progression to secondary nerve damage is a notable consequence [39,40,41]. Furthermore, inflammatory mediators released as a result of nerve injury as well as inflammation (i.e., bradykinin and adenosine triphosphate (ATP)) increase the amount of small-diameter sensory neurons positive for T-type Cav channels [42] that regulate peripheral sensory neuron excitability. This results in an increase in Cav currents in sensory neurons resulting in hypersensitivity [43,44,45,46]. Silencing of Cav3.2 has shown anti-nociceptive effects in animal models of neuropathic and inflammatory pain [43,47] and pharmacological inhibitors of T-type Cav channels has been shown to have analgesic effects in animal pain models [48,49,50].

Additionally, both animal and clinical electrophysiological studies have demonstrated altered firing properties of Aβ, Aδ and C fibers during the spontaneous activity associated with painful neuropathies [30,51,52]. Anticipated outcomes suggest that the occurrence of spontaneous burning or sharp pain is likely attributable to the spontaneous activity in C and Aδ fibers. Conversely, the spontaneous activity in Aβ fibers is expected to be associated with the manifestation of paresthesia and dysesthesia, which are commonly observed in neuropathies [33]. However, results of animal studies have shown that during the onset of pain, Aβ fibers (large-diameter myelinated afferents) comprise the majority of spontaneous activity. These afferents typically signal information about touch and vibration and directly contribute to spontaneous and evoked pain [53]. Thus, ectopic discharge in Aβ fibers, which in pathologic conditions become nociceptors themselves by undergoing a phenotypic switch, is a source of neuropathic pain [53]. Stimulation of previously injured Aβ fibers has also been shown to induce c-fos expression [54,55,56]. These findings provide further evidence for the idea that the activity in damaged Aβ fibers could be a dual contributor, both instigating pain and initiating central sensitization.

Studies have also found a mechanism of central sensitization that occurs via the unmasking of allodynia circuits [57]. In addition, glycine receptors in the dorsal horn may provide a therapeutic target. In [58], glycine blockade via an injection of a glycine receptor antagonist into the cisterna magna of a rat produced tactile allodynia through local circuits while selective inhibition of gamma isoform of protein kinase C and glutamate NMDA in the circuits disrupted them and prevented allodynia. It has also been shown that glycine inhibition can induce a dynamic mechanical allodynia resistant to morphine in cells lacking neurokinin 1 receptors [59]. These findings altogether suggest that in neuropathic pain, there exists a pathway that is then disinhibited, allowing Aβ afferents to access both deep and superficial dorsal horn laminae, resulting in mechanical allodynia [60].

To summarize, injury to every part of the pathway has been associated with neuropathic pain. Animal studies confirm multiple sites for generation of ectopic spontaneous activity along axonal projections and from the cell bodies within sensory ganglia [29,31,61]. Nerve injury affects a number of cells (immune cells, glia, and neurons) present at each of these anatomical levels, and each of these cells contribute to the development of neuropathic pain [62], communicating through gap junctions, synaptic transmission, and cell-to-cell signaling [63,64,65]. In situations of pathology, particularly after nerve injury, intensified communication among various cellular components may establish an environment conducive to heightened spontaneous activity [33]. Moreover, the alteration in characteristics of Aβ fibers (phenotypic switching) and their ectopic discharge play a role in the development of neuropathic pain and the accompanying sensory changes.

## 7. Ganglionic Sensitization and Phenotypic Change

**Figure 2:**
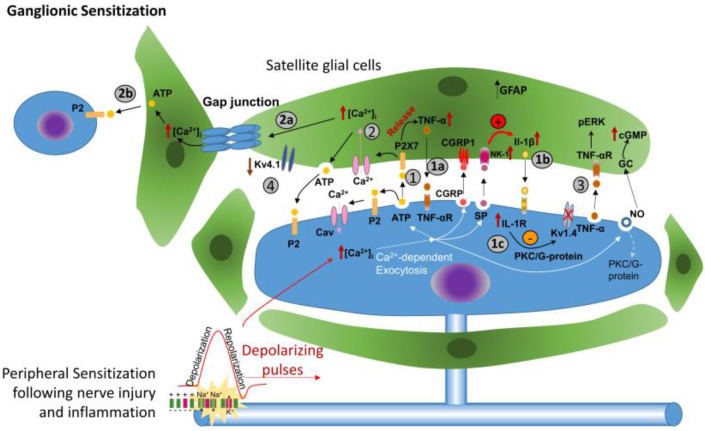
In ganglionic sensitization, (1) a peripheral nerve injury results in a somatic neurotransmitter release of CGRP, ATP, NO, and SP by the TG. These substances then bind to their respective receptors on the satellite glial cells (SGCs) that exist in the vicinity of TG neurons. The released ATP activates P2 × 7 receptors on SGCs, which results in the release of cytokines, such as TNF-α, which increases the neuron P2 × 3-mediated response. The exact mechanisms leading to SGC activation are not well understood; however, one of the suggested mechanisms is enhanced spontaneous neuronal activity. (1a,1b) SGC activation causes both increased production and release of the proinflammatory cytokines, resulting in the upregulation of IL-1R. (1c) IL-1β suppresses K^+^ currents, potentiating the excitability of nociceptors. (2) The release of ATP causes an increase in intracellular Ca^2+^, which (2a) propagates, and causes the further release of ATP in adjacent SGCs. (2b) The released ATP binds to purinergic receptors (P2), affecting the excitability of the neurons not directly affected by the injury. (3) TNF-α that was released by the SGCs induces increased phosphorylation of protein kinase. (4) Kv4.1 is downregulated, resulting in the depolarization and hyperexcitability of adjacent small-diameter neurons, producing spontaneous pain. Abbreviations: ATP = adenosine triphosphate, P2 = purinergic receptor, CGRP = calcitonin gene-related peptide, CGRP1 = calcitonin receptor, TNF-α = tumor necrosis factor alpha, TNF-αR = tumor necrosis factor alpha receptor, CGRP = calcitonin gene-related peptide, IL-1β = interleukin 1 beta, IL-1R = interleukin 1 receptor, pERK = phosphorylated extracellular regulated protein kinase, GC = guanylate cyclase alpha, cGMP = cyclic guanosine monophosphate, SP = substance P, PKC = protein kinase C. Figure reprinted with permission from author’s previous publication.

Hyperpolarization results from injury to a nerve because of subsequent inflammation that induces changes in the excitability and molecular composition of Nav, Kv, and Cav channels within the trigeminal ganglion (TG), as shown in Figure 2. Primary afferents then release pain-associated substances, such as substance P (SP), within the soma of neurons in the trigeminal ganglion.

Additionally, released nitric oxide (NO), nerve growth factor (NGF), or calcitonin gene-related peptide (CGRP) activate intra-ganglionic communication and thus contribute to the development and maintenance of trigeminal pathologic pain [66]. At the same time, peripheral inflammation increases neurokinin 1 (NK1) receptor expression in the satellite glial cells (SGCs) surrounding TG neurons. Activation of NK1 receptors on SGCs by SP released by TG neurons results in the increased production of interleukin one beta (Il-1β) in the SGCs, which is then released and activates its receptors on TG neurons. Activation of Il-1 receptors on TG neurons in turn leads to activation of protein kinase C/G-protein-coupled signaling that suppresses Kv currents in the TG neurons and therefore contributes to the potentiation of the neuronal excitability of TG neurons [67]. Additionally, peripheral nerve damage and inflammation result in a downregulation of a number of SGCs, inwardly rectifying potassium channels (Kir4.1), which could contribute to the depolarization of the neighboring small-diameter TG neurons, resulting in their hyperexcitability and spontaneous pain [67,68].

In summary, peripheral inflammation results in activation (depolarization) of trigeminal satellite glial cells by activation of NK1 receptors with the SP released from the TG nociceptor. Activated glial cells enhance production and secretion of pro-inflammatory cytokines (IL-1β, TNF-α) that activate their receptors on TG nociceptors, leading to hyperalgesia. Thus, SGCs can modulate the excitability of TG nociceptive neurons via IL-1β signaling, inducing membrane depolarization and increased expression of IL-1β receptor in neuronal body. Activation of the IL-1β receptor results in suppression of the voltage-gated potassium currents in the small-diameter trigeminal ganglion neurons, contributing to central sensitization responsible for hyperalgesia and allodynia [69]. SP activates and enhances the excitability of adjacent Aβ-TG neurons, leading to mechanical allodynia [70]. The cell–cell interactions among neurons and SGCs within the TG plays a pivotal role in regulating neuronal excitability [69] and potentially serves as a crucial factor in the development of orofacial sensory dysfunctions, such as ectopic hypersensitivity [71].

## 8. Central Mechanisms

Attacks of pain have been associated with increased activity in structures related to the sensory processing of pain (trigeminal nuclei, thalamus, and somatosensory cortices) as well as pain modulation, emotion, and memory (anterior cingulate cortex, insula cortex, prefrontal cortex, hippocampus, limbic system, and brainstem pain modulation system). These attacks have also been found to be associated with a reversible change in the functional connectivity of the frontal–limbic circuit as well as a reduction in gray matter in pain modulation and sensory, motor, and affective circuits.

On a molecular level, incoming sensory information from craniofacial tissues is transmitted and processed at the synapse with second-order neurons within the trigeminal sensory nuclear complex (TSNC), situated in the brainstem and upper cervical (C1-C2) spinal cord [72]. Within the TSNC, there exists the principal sensory nucleus (PrV), functioning as a relay station for non-noxious sensory signals, and the trigeminal spinal nucleus (SpV), serving as a relay station for the transmission of noxious orofacial sensory information [71]. The SpV is further functionally divided into the subnucleus oralis (Vi) in the rostral region, the subnucleus interpolaris (Vi), and the subnucleus caudalis (Vc) in the caudal region [73]. The Vi/Vc transition zone plays a role in processing deep tissue pain, integrating nociceptive orofacial pain, and contributing to the persistence of orofacial pain [74]. The Vc functions as a relay station for trigeminal nociceptive inputs originating in the face and oral cavity [75].

Nociceptive neurons of the orofacial region convey information to nociceptive neurons in the spinal subnucleus caudalis (Sp5C, medullary dorsal horn) and/or C1-2 cervical spinal cord [76]. The Sp5C and C1-2 are characterized by the presence of two types of neuron: wide dynamic range neurons (WDR) and nociceptive-specific neurons. WDR neurons respond to both noxious (via Aδ- and C-fibers) and non-noxious (via Aβ-fibers) stimulation within a large receptive field with a low mechanical threshold in the center, surrounded by an area of high mechanical threshold [77]. WDR neurons are found in superficial and deep lamina of Sp5C and C1-2 [78]. Two types of nociceptive-specific neurons respond exclusively to noxious stimuli: firstly, projection neurons that have long axons ascending to the thalamic nuclei and reticular formation, and secondly, neurons engaged in the function of local circuits within their immediate surroundings.

The lateral and medial pathways both ascend from the Sp5C. The lateral pathway conveys sensory/discriminatory information (i.e., exact location and intensity of the painful stimulus) from the Sp5C to the primary somatosensory cortex via the ventral posterior thalamic nucleus. The medial pathway conveys motivation and affective information from the Sp5C to the limbic system via the medial thalamic nucleus. Peripheral nerve injury and inflammation lead to hyperactivation of the nociceptive neurons in these two pathways, resulting in abnormal pain sensation in the region of injury.

Peripheral injury and/or inflammation leads to activation of intracellular signaling transduction pathways in nociceptor terminals leading to reduction of activation threshold and an increase in firing frequency (described in more details above). High-frequency impulses generated in the peripheral primary afferents are conducted to the central terminals of primary afferents resulting in neurotransmitter release. At the same time, there is an upregulation of receptors on the postsynaptic neurons in Vc and C1-C2. Therefore, increased activation of nociceptors (A-delta and C-fiber TG neurons) contributes to the sensitization of second-order (Vc and C1-C2) neurons and the development of spontaneous pain, hyperalgesia and allodynia.

### Role of Microglia

Microglia are the most abundant phagocytic cells in the CNS and are critical for production of cytokines, chemokines, and neurotropic factors. While the way in which microglia mediate central sensitization is not understood, their role in neuropathic pain is well studied. Microglia can activate M1 and M2, each with their own function. For example, M1 produces pro-inflammatory cytokines, such as TNF-α, IL-1β, IL-6, IL-8, and IL-17. They can disrupt the BBB, preventing resolution of inflammation, and when dysregulated, allow immune cells to access the CNS unchecked, sustaining the vicious cycle of chronic pain.

The nerve injury and subsequent inflammation contributes to astroglial and microglial cellular activation [79], resulting in modulation of SpVc/C1-2 neuronal excitability and neuroplastic change [65,80]. For example, following inferior alveolar nerve transection, astroglial cells become hyperactive and undergo morphological change by acquiring large soma and short processes [81]. Nerve injury results in increased expression of the transcription factor IRF8 (interferon regulatory factor 8), which in turn induces expression of interferon regulatory factor 5 (IRF5). IRF5 is responsible for direct transcriptional control of the P2 × 4 receptor, a non-selective cation channel activated by extracellular adenosine triphosphate (ATP) [82]. It binds to the promoter region of the P2rx4 gene and activates the de novo expression of the P2x4 receptor. In the presence of extracellular calcium, ATP stimulation of the P2x4 receptor results in structural changes such that the receptor opens a non-selective cation-permeable channel [83]. Influx of calcium into microglia activates p38-mitogen-activated protein kinase (MAPK), which results in release of brain-derived neurotrophic factor (BDNF) [84,85,86].

The release of BDNF by microglia plays a crucial role in the onset of neuropathic pain and the initiation of central sensitization [87,88,89]. This is because BDNF either alleviates the inhibition within the dorsal horn nociceptive network [86] or enhances excitatory synaptic transmission [84]. The released BDNF binds to its receptor, located on dorsal horn neurons, and downregulates potassium-chloride transporter (KCC2) through tropomyosin-related kinase B, resulting in an increase of chloride concentration in dorsal horn neurons (Tsuda 2016) and thus disrupting intracellular chloride homeostasis. The communication between microglia and neurons, leading to disinhibition, amplifies excitatory synaptic transmission and alters the functioning of the nociceptive network [85].

At the same time, gamma aminobutyric acid (GABA) and glycine released from the activated central terminals of peripheral nociceptors activate gamma aminobutyric acid receptor alpha (GABA-A), which upon activation selectively conducts chloride through its pores, resulting in depolarization of second-order neurons [90]. Additionally, synaptic transmission in the Sp5C/C1-2 is modulated by the astroglial glutamine-glutamate shuttle [91,92]. Activated astroglia release glutamine, which is transported out and taken up by presynaptic terminals via glutamine transport. Glutamine in the presynaptic terminal of sensory neuron is transformed into glutamate, which increases the glutamate release from the presynaptic terminal [78]. Therefore, astroglial activation may contribute to central sensitization of nociceptive neurons in rat Sp5C (medullary dorsal horn), resulting in orofacial hyperalgesia and/or allodynia [78]. Following peripheral nerve injury, there is an initial occurrence of p38 mitogen-activated protein kinase (p38-MAPK) expression in activated microglia, followed by the subsequent activation of nuclear factor kappa B (phosphor-NF-kB) in astrocytes within the medullary dorsal horn. Both these events play a crucial role in the development of trigeminal neuropathic pain [81,93].

To summarize, phenotypic changes of lamina I neurons, including A-δ and C-fibers, result in the following: (1) an increased output in response to nociceptive stimuli, (2) the relay of innocuous mechanical input, and (3) the generation of spontaneous bursts of activity [86,94,95,96]. These changes in the cellular properties of lamina I neurons constitute the foundation of the primary manifestations of neuropathic pain, encompassing allodynia, hyperalgesia, and spontaneous pain [85].

## 9. Central Sensitization

**Figure 3:**
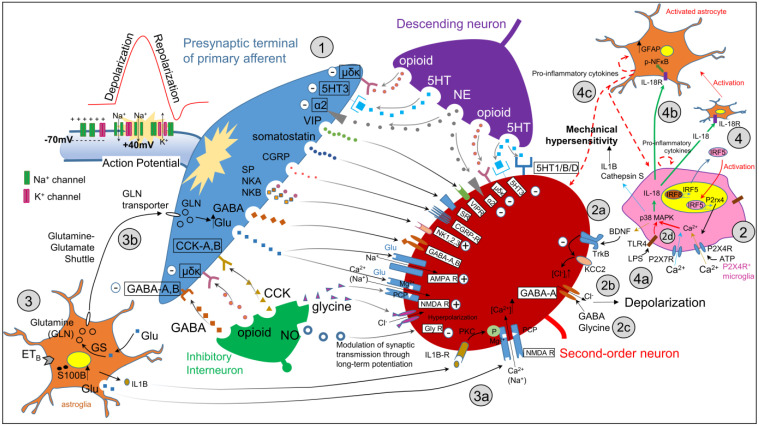
Central sensitization and modulation: (1) An action potential generated in the nociceptor peripheral terminal is conducted along the peripheral process to the central process in the trigeminal sensory nuclear complex, where it depolarizes the presynaptic terminal of the primary afferent. A number of neurotransmitters are released from the central terminal of the depolarized primary afferent to the synaptic cleft. These include glutamate (glu), gamma aminobutyric acid (GABA), substance P (SP), neurokinin A (NKA), neurokinin B (NKB), calcitonin gene-related peptide (CGRP), somatostatin, vasopressin inhibitory protein (VIP), and other peptides that bind to the second-order neuron, resulting in its depolarization. This leads to its activation. The primary afferent’s presynaptic terminal communicates with the second-order neurons and interneurons, descending neurons, and microglia as well as astroglia. (2) Peripheral nerve injury causes the microglia to upregulate P2x4, and its activation causes an extracellular influx of Ca^2+^, activating p38 MAPK. (2a) This causes microglia to release BDNF, which resulting in the inhibition of KCC2, (2b) disruption of Cl- homeostasis, and disinhibition of the second-order neurons. (2c) GABA/glycinergic inhibitory tone is lost, and (2d) P2 × 7-p38 MAPK signaling results in IL-1β and cathepsin S release. (3) In the astroglia, IL-1β is released, activating PKC, phosphorylating the NDMA receptor subunit, NR1, and (3a) causing the intracellular release of calcium. Therefore, IL-1R-mediated signaling may regulate NMDA receptor function and increase synaptic strength via post-translational phosphorylation and contribute to persistent pain and pain hypersensitivity (3a). Additionally, the glutamine–glutamate shuttle modulates synaptic transmission in the subnucleus caudalis and C1-C2. Following nerve injury, activated astroglia release glutamine, which is transported out and taken up by presynaptic terminals via glutamine transport (3b). In the presynaptic terminal, glutamine is converted to glutamate, which increases the glutamate release from the presynaptic terminal, contributing to central sensitization of nociceptive neurons in the medullary dorsal horn. Astrocytes respond to nerve injuries by upregulating production of a calcium-binding protein (S100β). Elevated levels of S100B have been found in the spinal cord following hind paw inflammation, L5 spinal nerve ligation, and spinal cord injury as well as the rostral ventromedial medulla after infraorbital nerve injury. Hypersensitivity caused by nerve injury is attenuated in S100β knockout animals and enhanced in animals expressing S100β. (4) Following nerve injury, astrocytes increase ETB expression; ETB receptors bind endothelins, resulting in the activation of astroglia. (4a) In a possible mechanism, microglia are activated by LPS binding to TLR4. (4b) Release of IL-18 results in NFkB phosphorylation, activating astrocytes. (4c) Proinflammatory molecules are released, in part, because of the activation of microglia and astrocytes, which sensitizes second-order neurons further. Abbreviations: VIP = vasopressin inhibitory protein; glu = glutamate; NE = norepinephrine; SP = substance P; NKA = neurokinin A; NKB = neurokinin B; GFAP = glial fibrillary acidic protein; cck = chemokine; GABA = gamma aminobutyric acid; CGRP = calcitonin gene-related peptide, 5HT = serotonin; MAPK = mitogen-activated protein kinase; IRF = interferon; IL = interleukin; NMDA = N-methyl-D-aspartate receptor; GLN = glutamine; BDNF = brain-derived neurotrophic factor; PCP = phencyclidine; KCC2 = potassium chloride transporter; NO = nitric oxide; TLR4 = toll-like receptor 4; ETB = endothelin B receptor.

Central sensitization is defined as the enhanced excitability of central nociceptive pathways post tissue injury and inflammation. It is marked by both magnified responses to nociceptive stimuli and perceived pain spread to neighboring areas. Additionally, central sensitization has been implicated in the development of allodynia and other sensory changes, prompted by continuous activity of primary afferents via ‘wind-up’. This phenomenon is one type of central sensitization in which amplified response occurs when there are repeated primary nociceptive afferent inputs, resulting in increasingly sensitized CNS neurons. Central sensitization occurs when there is hypersensitivity that explains the rise in pain intensity and its extension to neighboring structures in individuals experiencing severe facial pain, interactions of which can be seen in Figure 3.

Besides the initial input from primary afferent nociceptors, the involvement of glial cells and the influence of extracellular adenosine 5′-triphosphate (ATP) on various purinergic receptors have been demonstrated in contributing to central sensitization in the medullary dorsal horn (MDH) [97]. Of the seven P2X receptors present in the spinal cord, P2 × 7 and P2 × 4 receptors located primarily in microglia have been implicated in chronic inflammatory and neuropathic pain [90,98,99,100]. Astrocytes have a close anatomical relationship with neurons, and the interactions between the astrocytes and neurons contribute to the mechanisms of synaptic plasticity and central nervous system disorders. The astroglial glutamate–glutamine shuttle has been shown to play a role in central sensitization of nociceptive neurons in MDH [92]. The glutamate–glutamine shuttle is a key function of astroglia, in which astroglia uptakes excessive extrasynaptic glutamate and uses glutamine synthetase (GS) to produce and release glutamine. The released glutamine is taken up by the central terminals of presynaptic nociceptive neurons and is converted to glutamate, replenishing the glutamate supply [101]. Inhibition of GS activity in astroglia has been shown to attenuate central sensitization induced in nociceptive (wide dynamic range and nociception-specific) neurons in trigeminal subnucleus caudalis [92]. Presynaptic glutamine uptake has been shown to be essential for the manifestation of central sensitization and validates the involvement of astroglia, glutamine, and presynaptic modulation of glutamate release in central sensitization initiation [102].

As shown in Figure 1, nerve injury results in a decrease of GLT-1 expression in astrocytes [103] resulting in a buildup of glutamate concentration in the synaptic cleft and development of hyperexcitability and hyperalgesia [91]. Astrocytes are also responsible for removal of gamma-aminobutyric acid (GABA) (an inhibitory amino acid transmitter released from GABAergic presynaptic terminals). Facial inflammation has been shown to increase expression of GABA transporter (GAT) in the spinal trigeminal nucleus of the injured side [104], which may facilitate GABA removal and result in a reduction of GABAergic inhibition, consistent with central hyperexcitability and hyperalgesia [91].

Furthermore, astrocytes may affect neuronal activity via calcium-dependent exocytotic release of gliotransmitters such as glutamate [105] and release of the NMDA receptor co-agonist, D-serine [106,107]. Thus, glial-derived glutamate may affect hyperexcitability by activating extrasynaptic NMDA receptors [108].

It is important to recognize the mounting evidence suggesting that untreated, persistent pain induces sensitization and brings about changes in both the peripheral and central nervous systems, laying the groundwork for the establishment of chronic pain. The progression following nerve injury or extensive tissue damage emphasizes the need for early treatment, especially when prevention is not always feasible.

## 10. Descending Modulation of Orofacial Pain

The descending system modulates neuronal activity by acting on nociceptive neurons at each level of the ascending pain pathway. The primary pathway for modulating pain involves a descending circuit that begins in the midbrain periaqueductal grey (PAG) and extends to the rostral ventromedial medulla (RVM), where it works to suppress ascending nociceptive transmission at the spinal cord dorsal horn [109]. Anti-nociception is achieved by activation of neurons projecting from the PAG to RVM [110] leading to changes in the excitability of Vc nociceptive neurons carrying nociceptive signals to higher levels of CNS.

Descending projections have also been found to modulate pain from the A11 and paraventricular nucleus as well as the lateral, perifornical, and retrochiasmatic areas in the hypothalamus modulate pain via projections descending to the Sp5C [111].

There are two functionally distinct classes of RVM neurons: ON cells, which increase neuronal activity following peripheral afferent activation, and OFF cells, which decrease neuronal activity following peripheral afferent activation [110]. The OFF cells serve as the principal output neurons in the descending PAG-RVM pathway, projecting to the spinal cord [112]. In contrast, the ON cells function as inhibitory GABAergic interneurons within the RVM [109]. The analgesic impact resulting from the activation of the descending output from the PAG is realized through the inhibition of ON cells and the enhancement of OFF cell activity, facilitated by opioid signaling [110].

Opioids have been shown to directly inhibit neurons and produce analgesia when injected into the PAG and RVM [113]. There is a hypothesis suggesting that opioids induce the descending inhibition of nociceptive transmission (analgesia) through a mechanism known as ‘GABA disinhibition’. In this process, opioids stimulate descending projection neurons (OFF cells) by laterally inhibiting neighboring GABAergic neurons (ON cells) [109]. Additionally, it has been shown that µ-opioids and cannabinoids suppress excitatory glutamatergic transmission in the PAG and RVM, suggesting that opioid signaling in the PAG and RVM is complex and affects physiologically opposite responses, inhibitory GABAergic and excitatory glutamatergic activities [114,115]. Therefore, the manifestation of pro- and anti-nociception may be characterized by an equilibrium between inhibitory and excitatory processes, contingent upon the timing relationship between glutaminergic and GABAergic synaptic transmission [109].

Serotonergic RVM neurons projecting to Vc and C1-C2 can change the excitability of nociceptive neurons in Vc and C1-C2. Serotonin (5-HT) released from these serotonergic neurons can exert either inhibitory or excitatory effects in the Vc and C1-C2, depending on the receptor subtype. There are several different 5-HT receptor subtypes expressed in the central terminals of nociceptive primary afferents. These include inhibitory 5-HT receptors 5-HT1A, 5-HT1B, and 5-HT1C as well as excitatory 5-HT receptors 5-HT2A, 5-HT3 and 5-HT4 [71]. Activation of inhibitory 5-HT1B receptor on the presynaptic terminal modulates nociceptive neuronal activity by reducing glutamate release from primary afferent terminals in Vc and C1-C2 [116]. The inhibitory receptors 5-HT1A are expressed by second-order neurons. Activation of these receptors results in suppression of nociceptive neuron activity. The central terminals of nociceptive primary afferents and GABAergic interneurons in the Vc and C1-C2 express an excitatory 5-HT receptor 5-HT7 [117]. Activation of 5-HT7 5-HT receptors stimulates the release of GABA, resulting in suppression of the excitability of these neurons and therefore inhibition of mechanical hypersensitivity following capsaicin treatment in mice [118]. Moreover, activation of the 5-HT2A receptor in the Sp5C of rats induced phenotypic changes in PKC interneurons, inducing mechanical allodynia [119].

Noradrenaline (NA) plays a role in inhibitory descending pain modulation. Neurons from the noradrenergic locus coeruleus and Kolliker–Fuse nuclei project directly to the dorsal horns of the medulla and spinal cord [120,121]. NA released by these neurons activates its receptor (α2-adrenergic receptor) in the central terminals of primary nociceptive neurons and inhibits pre- and post-synaptic nociceptive transmission by suppressing the release of excitatory neurotransmitters (e.g., glutamate) from primary afferents. Additionally, NA exerts the anti-nociceptive effects by activating the α2-adrenergic receptor on second-order neurons. It inhibits the hyperexcitability of second-order nociceptors by binding to α1-adrenergic receptor in GABAergic interneurons and leading to their depolarization.

In short, the precise mechanisms of descending pain modulation within the medullary system are not fully understood. They entail an intricate interplay between opioid, 5-HT, NA, and GABA signaling within second-order nociceptive neurons in Vc and C1-C2. The direction of descending pain modulation, whether inhibitory or facilitatory, is determined by the delicate balance among these signaling pathways [71].

## 11. Conclusions

PTTN is a type of neuropathic pain that occurs following traumatic injury to a nerve in the trigeminal pathway. Several etiologies are possible, from dental procedures to motor vehicle accidents to local anesthetic injections. This damage results in altered electrical activity via the release of pro-inflammatory mediators and phenotypic changes. Cytokines and chemokines are dysregulated in neuropathic pain and may be the targets of treatment. Dysregulated voltage-gated sodium, potassium, and calcium channels also play a role in the development of trigeminal neuropathy. Peripheral and central mechanisms work in tandem to trigger and maintain neuropathic pain. More studies are needed to understand whether mechanisms of PTTN differ from current animal models of neuropathic pain.

## Data Availability

Not applicable.
